# P02.04. Internet survey confirms strong interest in Yoga among fibromyalgia patients

**DOI:** 10.1186/1472-6882-12-S1-P60

**Published:** 2012-06-12

**Authors:** J Carson, R Bennett, K Jones, S Mist

**Affiliations:** 1 Oregon Health & Science University, Portland, USA

## Purpose

Studies in circumscribed clinical settings have reported that yoga has been adopted by fibromyalgia (FM) patients of many cultural backgrounds. However, yoga styles vary in the methods they emphasize, and it is unclear from existing studies which types of practices FM patients are typically engaging in, and the extent to which they experience yoga as helpful or not. The purpose of this study was to survey FM patients in many different regions to inquire about their engagement in various yoga practices, perceived benefits, and obstacles to further practice.

## Methods

We conducted a 16 question internet survey of persons self-identified as FM patients who were subscribers to electronic newsletters on the topic of FM. Respondents (N=2543) replied from all 50 U.S. states and also Canada, Australia and the United Kingdom.

## Results

The average age of respondents was 57 years, 96% were female, and average time since diagnosis was 13 years. Of these, 80% had considered trying yoga and 58% had attended ≥1 yoga class. Their classes typically focused almost exclusively on yoga poses, with minimal training in meditation, breathing techniques or other practices. The most commonly cited benefits were reduced stiffness, relaxation, and better balance. The most frequently cited obstacles were fear that the poses would cause too much pain, concerns about the poses being too physically demanding, or not being able to do the poses correctly.

## Conclusion

These findings confirm strong interest in yoga across a geographically diverse range of FM patients. However, concerns about yoga-induced pain and yoga poses being too difficult are common reasons that FM patients do not engage in yoga exercises. This study supports the need for yoga programs tailored for FM that include modification of poses to minimize aggravating movements, and substantive training in meditation and other yoga-based coping methods to minimize pain-related fear.

